# The acute effect of verbal instructions on performance and landing when dropping from different heights: the ground reaction force-time profile of drop vertical jumps in female volleyball athletes

**DOI:** 10.3389/fspor.2024.1474537

**Published:** 2024-10-24

**Authors:** Stefano La Greca, Gaetano Antonacci, Stefano Marinelli, Pierangelo Cifelli, Riccardo Di Giminiani

**Affiliations:** Department of Biotechnological and Applied Clinical Sciences, University of L’Aquila, L’Aquila, Italy

**Keywords:** plyometric, motor learning, explosive strength, exercise physiology, sport

## Abstract

**Introduction:**

The drop vertical jump (DVJ) is extensively utilized for conditioning and evaluating physical performance, as well as reducing the likelihood of injury by enhancing joint stability through the coactivation of muscles acting on the joint. The execution of DVJ can be controlled by verbal instructions and evaluated by the vertical ground reaction force (vGRF)-time profile.

**Methods:**

Our hypothesis was that varying verbal instructions could have an impact on the DVJ's parameter, thereby optimizing vertical performance and minimizing the impact during landing in young female volleyball players. Sixteen female volleyball players volunteered to participate in this study (age: 21.3 ± 2.6 years; stature: 1.66 ± 0.1 m; body mass: 62.0 ± 8.1 kg and BMI: 22.2 ± 1.8). They executed DVJs following verbal instructions ranging from “jump as high as possible” (1A), “jump as quickly as possible” (2A), “jump as high as possible and during the landing attempt to dampen the impact at ground contact” (1B), and “jump as high as quickly as possible and during the landing attempt to damp the impact at ground contact” (2B). The reactive strength index (RSI), vGRF (1st and 2nd peaks), and flight time (FT) were evaluated.

**Results:**

The verbal instructions 1A improved the FT and the first peak of the vGRF (*P* < 0.05), whereas 2A the RSI (*P* < 0.05). On the contrary, “the damping” required in the 1B, and 2B verbal instruction reduced the 2nd peak of vGRF (*P* < 0.05), without altering the task required during the jump (*P* < 0.05).

**Discussion:**

The instructions provided for the final landing (the second peak of vGRF) have the potential to enhance safety by reducing the peak of vGRF without affecting the performance required during the jump. When designing DVJ training, coaches or kinesiologists must consider the use of verbal instructions to induce specific adaptation over time.

**Clinical relevance:**

The present study supports the use of specific verbal instructions to reduce impact forces in landing and injury risk.

## 1 Introduction

Verbal instructions represent a powerful stimulus capable of influencing higher cognitive processes such as behavior, attention and learning. Indeed, verbal instructions have been reported to have a major impact on movement and posture control, highlighting their importance in improving movement execution and suggesting the existence of an influence of the mind on the body (1, 2). Particularly, it has been (3–6), suggested that verbal instructions could induce a focus on execution task accelerating the learning process so that an advanced level of performance is achieved sooner (7). This information becomes relevant in the sports context, highlighting the importance of verbal instructions in improving performance. In fact, in nearly every training situation that involves learning motor skills, athletes are instructed to follow the correct movement pattern or technique. According to the “constrained action hypothesis” proposed by Wulf (7, 8), instructions appear to direct the attention towards correct movements, triggering an automatic mode of motor control guided by unconscious processes (operating at an automatic level) and achieving the desired outcome. Moreover, when comparing conditions with instructions to those without them, the muscular activity for the identical performance outcomes is significantly diminished, both in the muscles of agonists and antagonists (9). This indicates a higher degree of movement efficiency in terms of the recruitment of muscle fibers, as well as enhanced inter-muscular coordination. It appears that the instructions increase movement efficiency and reduce noise in the motor system, which hampers fine control and makes the outcome of the movement less effective (8).

In this context, plyometric exercises have been a subject of great interest in sports exercise research for over 60 years (10). The plyometric jumps or drop vertical jump (DVJ) are exercises typically employed in explosive strength training (11–13) to improve vertical jump performance (14, Bosco and Komi 1979b, 15, 16), running speed, and acceleration (17). Moreover, DVJ exercises are extensively utilized in diverse settings, including but not limited to preventive measures, which aim to reduce the likelihood of injury to the Anterior Cruciate Ligament (ACL) due to non-contact mechanisms (18, 19); screening assessments, which assess susceptibility to ACL rupture (3, 6, 19); and as adapted exercises, for ankle stability (20). One of the most renowned techniques employed in DVJ is the Depth Jump (DeJ) and the Drop Jump (DJ). The concept of DeJ was proposed by Verkhoshansky's initial studies (21), where in a subject standing on a box performs a free fall and, subsequent to ground contact, must attain a greater jump height, without any restrictions on the contact time or range of motion (ROM) of the lower limb joints (10, 22). In the DJ, according to (Bosco and Komi 1978, 15, 23), the subject must accelerate rapidly after contact with the ground, thereby enhancing the reactive strength index (RSI) (24).

In the training process, these two exercises have often been confused and used without consideration. Nonetheless, the distinction between the two-modality lies precisely in the execution mode; ground contact time and emphasis on ROM degrees serve as the primary distinguishing factors underlying the two plyometric jump modes (22). The effectiveness of DVJ on vertical performance appears to be superior to other types of jumps (25, 26).

During DVJ exercises, the interaction between the body and the environment is characterized by a significant shift in reaction forces (27) and a muscle action called the stretch shortening cycle (SSC) is performed, in which the stretching force is imposed on the neuromuscular system, preparing the body to counteract the effects of gravity. In the active braking phase of the SSC, the impact loads and the nature of the stretches are determined by the drop height (15, 26, 28, 29), and regulated by afferents from proprioceptive receptors that are integrated by the central nervous system (30, 31). Hence, during the propulsion phase, the power production could be enhanced by the neural potentiation (26, 30, 32, 33) occurring at individual dropping heights or stretch loads (28, 29). On the contrary, when an excessively muscle forces due to high dropping height (high stretch load) is generated, the Golgi Tendon Organs (GTO) should detect the tension, and a reflex inhibition could be elicited on the same muscle in an attempt to reduce the high ground reaction forces preserving the musculotendinous or joint integrity (34, 35). Considering these neuromechanical characteristics, the DVJ is an highly effective technique for enhancing not only the performance of vertical jump-related tasks but also the functional characteristics of numerous sports that necessitate reactive strength abilities, such as soccer, sprinting, and handball (13, 31, 36, 37; Montoro-Bombú).

The impact of verbal instructions in DVJ was investigated by Khuu et al. (5), who examined different verbal instruction effects and found that contact was stiffer with a decrease in jump height when asked to “reduce contact times” in comparison to “reach maximum height.” The results were similar to those reported by Yokoyama et al. (6), who studied two different verbal instructions, “high jump” and “quick jump”, and found that the second verbal instruction increased the vGRF during the braking phase.

The studies reported in the literature examined the impact of verbal instructions on DJ when the subjects were dropped from a single drop height (4–6) but not during different drop heights. Another important methodological aspect that has not been considered in the literature regards the lack of specific verbal instructions capable of reducing the vGRF during the landing (2nd peak) without compromising the DJ performance. This study investigated the effect of four different verbal instructions on some parameters of the vGRF-time profile recorded during the DVJ (FT, 1st and 2nd vGRF, and RSI) and their possible interactions with different drop heights (20, 30, and 40 cm) in young female volleyball players. We hypothesized that different verbal instructions could influence the DVJ's parameter, which would in turn optimize the vertical performance and reduce the impact during the landing in young female volleyball players.

## 2 Materials and methods

### 2.1 Study design and participants

A single-group repeated-measures study design was used in which the kinetics and kinematics parameters of the vGRF-time profile (i.e., the 1st and 2nd vGRF peak, FT, and RSI) were considered for analysis. The independent variables were the different drop heights and the verbal instructions. The measurements were conducted in the biomechanics laboratory of the university, and each participant accessed the laboratory on two separate occasions, with a minimum of one day between each visit. The ethical standards of the Declaration of Helsinki were followed, and the participants provided written informed consent before the measurements. The study was approved by the Internal Review Board of the University (no. 33/2022).

Sixteen female volleyball players voluntarily took part in this study (age: 21.3 ± 2.6 years; stature: 1.66 ± 0.1 m; body mass: 62.0 ± 8.1 kg and BMI: 22.2 ± 1.8). All the participants were athletes competing at regional level and the drop jump exercises were included in their weekly training at least once within the three-training session per week. They declared that they had not experienced any injuries or musculoskeletal pain in the past 12 months. The sample size was computed *a priori* by means of statistical software for power analysis (G*Power 3.1.9.4, Heinrich Heine-Dusseldorf University, Düsseldorf, Germany). The computation was performed in relation to the study design (F tests—two way ANOVA: Repeated measures, within factors), setting the effect size, and using the protocol for a power analysis (test attributes, effect size [0.30], *α* = 0.01, power [1−*β*] = 0.95, total sample size *n* = 15 participants).

### 2.2 Test procedure and data collection

During the first lab visit, the participants familiarized themselves with the experimental procedure. They warmed-up for approximately 15 min (8 min run on a treadmill at a speed of 6 km/h, 2 min dynamic stretching, and 5 min mono- and bipodalic stance leaps) and then executed DVJs in a random manner, for each drop height of 20 cm, 30 cm, and 40 cm. The DVJs were performed by dropping from a box and immediately following ground contact, performing a maximal vertical jump. The exercise was concluded with a second landing. To ensure the correct execution of the jumps, the subjects were instructed with explicit verbal instructions, specific to plyometric jumps: “Keeping your hands on your hips and with the foot you feel most comfortable, step off the box, land on two feet, and jump straight up with maximum effort. The box was of different heights (20, 30, and 40 cm) and was positioned beside a force plate (D-Wall, TecnoBody s.r.l., Bergamo, Italy; sampling rate 100 Hz) into which the participants performed DVJs.

**Figure 1:**
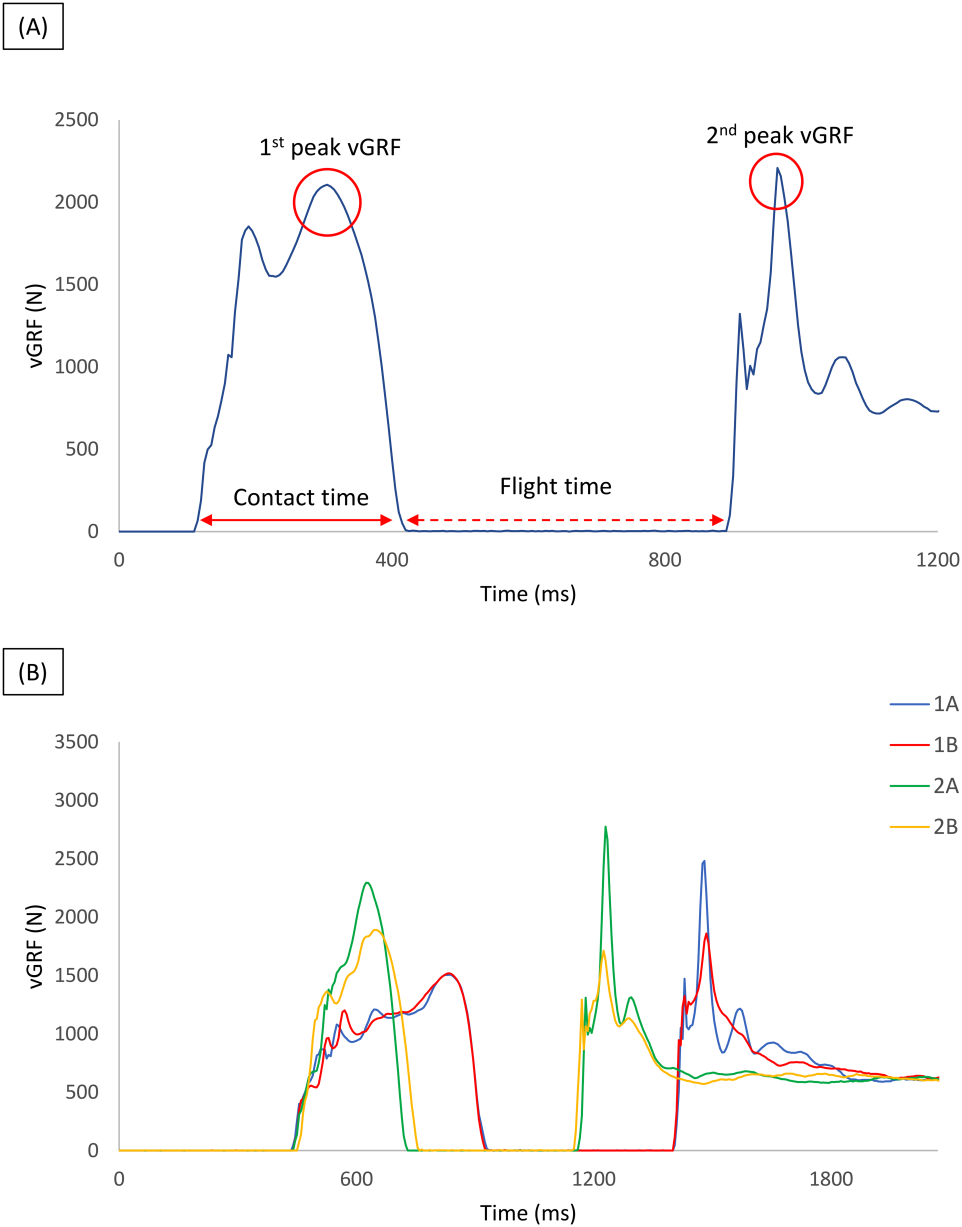
**(A)** vGRF-time profile and the underlined parameters (1st peak vGRF; 2nd peak vGRF; contact time; flight time); **(B)** representatives force-time profiles performed with the different verbal instructions (**1A**: “jump as high as possible”; **2A**: “jump as quickly as possible”; **1B**: “jump as high as possible and during the landing attempt to dampen the impact at ground contact”; **2B**: “jump as high as quickly as possible and during the landing attempt to dampen the impact at ground contact”). vGRF, vertical ground reaction force.

During the second laboratory visit, prior to the measurements, the participants underwent a 15-min warm-up, identical to that of the first laboratory visit. The vGRF-time profile was recorded during each DVJ and the following parameters were taken into account for analysis: FT (the time spent in air between the final push-off phase and before the initial ground contact at landing), the 1st peak (the peak of vGRF produced during the braking-propulsion phase), the 2nd peak (the peak of vGRF produced during the landing phase), and the RSI [the ratio between the FT divided by the contact time, the time between the feet’ ground contact and the take-off, (38)] (Figure 1A).

Each participant executed a total of 24 DVJs; two trials were averaged for analysis for each jump height (20, 30, and 40 cm) and for each verbal instruction (4 conditions) in a random order. The recovery between the several trials was about 40–60 s, and the verbal instructions included the following: 1A, 2A, 1B, and 2B. In the 1A condition, participants were instructed to “jump as high as possible” to achieve the maximum jump height whereas in condition 2A, the emphasis was placed on minimizing ground contact time by instructing the participants to “jump as quickly as possible”. In the other two conditions, the emphasis was placed on the landing phase, with the aim of reducing the vGRF at landing (the second peak). Specifically, in the condition 1B, the subjects were instructed to “jump as high as possible and during the landing attempt to dampen the impact at ground contact,” whereas in the 2B condition, the subjects were instructed to “jump as high as quickly as possible and during the landing attempt to dampen the impact at ground contact” (Figure 1B).

### 2.3 Statistical analysis

The analysis was executed using the statistical software XLSTAT 2013.2.07 (Addinsoft, SARL, New York). The values of the dependent variables exhibited a normal distribution, as demonstrated by Shapiro-Wilks's *W* test. The dependent variables were analyzed using a mixed model repeated measures two-way ANOVA with a compound symmetry working covariance matrix, and the Bonferroni correction adjusted the *p*-values according to the number of comparisons.

In a comparable group (29) the intra-session reliability of the measurements was quantified using the intra-class correlation coefficient (ICC of single measures) (39). The ICC values below 0.50 are classified as “poor”, those between 0.50 and 0.69 are classified as “moderate”, those between 0.70 and 0.89 are classified as “high”, and those above 0.90 are classified as “excellent”. The effect size of the ANOVA analysis was determined by using partial eta squared (*η*_p_^2^) (40, 41) with values that were considered small (*η*_p_^2^ = 0.01), moderate (*η*_p_^2^ = 0.06) or large (*η*_p_^2^ = 0.14). The effect size of the contrasts was determined using Hedges’ g, which was considered to be small at *g* < 0.5, moderate at 0.5 < g < 0.8, and large at *g* > 0.8. The significant level was set at *α* = 0.05.

## 3 Results

The intrasession reliability for all drop height measurements was “excellent” (ICC > 0.90).

**Figure 2:**
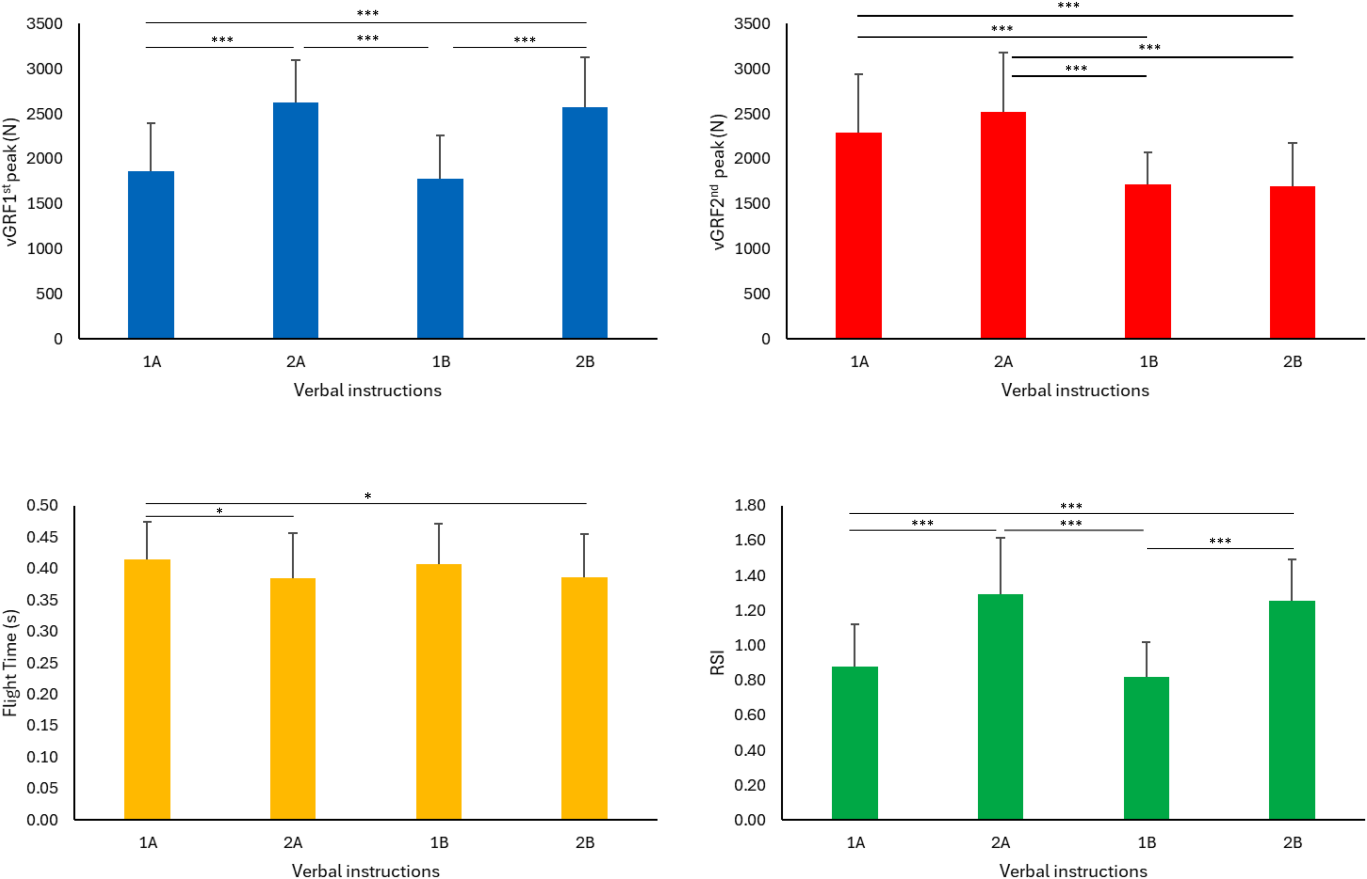
Mean values and standard deviation of the different parameters are reported. *Significant difference: *P* ≤ 0.05; ***significant difference: *P* < 0.01. vGRF, vertical ground reaction force; RSI, reactive strength index.

**Figure 3:**
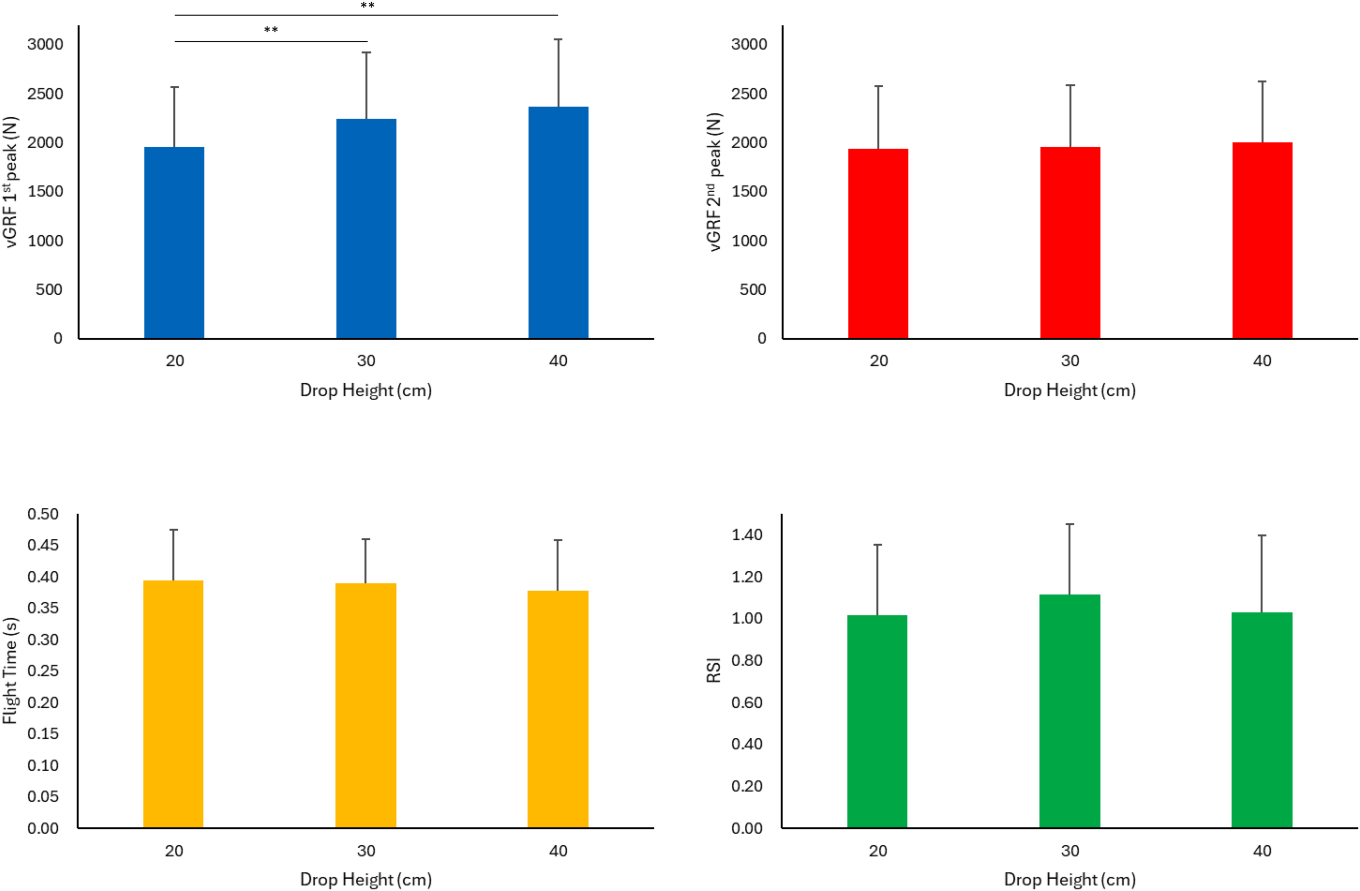
Mean values and standard deviation of the different parameters are reported. **Significant difference: *P* ≤ 0.05. vGRF, vertical ground reaction force; RSI, reactive strength index.

The flight time during the drop jump depended on the drop height [F_(2.180)_ = 3.419; *P =* 0.035; *η*_p_^2^ = 0.04] and verbal instructions *[*F_(3.180)_ = 3.182; *P* = 0.024; *η*_p_^2^ = 0.05], whereas the interaction between the drop height and verbal instructions did not affect the flight time [F_(6.180)_ = 0.603; *P* = 0.728; *η*_p_^2^ = 0.02]. Significant contrasts were observed between the verbal instructions 1A and 2A (*P* = 0.038, ES = 0.46) and between 1A and 2B (*P* = 0.050, ES = 0.44) (Figure 2), whereas the drop heights did not exhibit any differences (*P* > 0.05) (Figure 3).

The vGRF during braking phase (1stpeak) depended on drop height [F_(2.180)_ = 20.036; *P =* 0. 0001; *η*_p_^2^ = 0.18] and verbal instructions *[*F_(2.180)_ = 70.152; *P* = 0.0001; *η*_p_^2^ = 0.54]. Vice versa the interaction between drop height and verbal instructions was not significant [F_(6.180)_ = 0.465; *P* = 0.833; *η*_p_^2^ = 0.01]. Significant contrasts were found between the verbal instructions 1A and 2A (*P* < 0.001, ES = 1.54), 1A and 2B (*P* < 0.001, ES = 1.32), 2A vs. 1B (*P* < 0.001, ES = 1.77), and 1B vs. 2B (*P* < 0.001, ES = 1.5) (Figure 2). The drop height from 20 cm to 30 cm and from 20 cm to 40 cm were significant (*P* = 0.009, ES = 0.49; *P* = 0.001, ES = 0.66) (Figure 3).

The verbal instructions affected the landing phase (2ndpeak of vGRF) [F_(3.180)_ = 58.659; *P* = 0. 0001; *η*_p_^2^ = 0.49]. The drop height [F_(2.180)_ = 0.318; *P* = 0.728; *η*_p_^2^ = 0.003] or the interaction between drop height and verbal instructions [F_(6.180)_ = 0.330; *P* = 0.920; *η*_p_^2^ = 0.01] did not affect the landing phase significantly. Significant contrasts were found between the verbal instructions 1A and 2B (*P* < 0.001, ES = 1.06), 1A and 1B (*P* < 0.001, ES = 1.12), 2A and 2B (*P* < 0.001, ES = 1.42), and 2A vs. 1B (*P* < 0.001, ES = 1.51) (Figure 2).

The drop height [F_(2.180)_ = 3.636; *P* = 0. 028; *η*_p_^2^ = 0.04] and verbal instructions [F_(3.180)_ = 69.800; *P* = 0.0001; *η*_p_^2^ = 0.54] affected RSI. On the contrary, their interaction was not significant [F_(6.180)_ = 0.674; *P* = 0.870; *η*_p_^2^ = 0.02]. Significant contrasts were found between the verbal instructions 1A and 2A (*P* < 0.001, ES = 1.46), 1A and 2B (*P* < 0.001, ES = 1.59), 2A and 1B (*P* < 0.001, ES = 1.77) and 1B vs. 2B (*P* < 0.001, ES = 2.02) (Figure 2) but not among the different drop heights (Figure 3).

## 4 Discussion

This study investigated the influence of verbal instruction on some biomechanical parameters that are commonly included in DVJ performance when performed from different drop heights. Indeed, it is known that verbal stimuli can influence behavior and motor control through the involvement of neural, cognitive and motivational mechanisms, with effects on sports performance. Our findings confirm the hypothesis that verbal instructions and different drop heights can influence the performance of the DVJ. On the contrary, the interaction between drop height and verbal instructions was not significant at any of the analyzed parameters.

The verbal instructions have shown to improve the FT when asked to jump as high as possible (1A), and the jump performed is a DeJ. However, when asked to jump as fast as possible (2A), the RSI increased due to stiff contact, and the jump performed is a DJ. Based on the previous literature, these results are not completely unexpected, in fact, some studies have shown how external feedback (i.e., video feedback showing correct execution techniques) (4) or verbal instructions (ask to “minimize contact time” or “maximize jump height”) (5, 6, 42) can influence the typical parameters of DVJ performance (i.e., CT, RSI, Power, vGRF, FT, maximum CoG height) and the joints kinematics of lower extremity (hip, knee, and ankle) (4–6, 42). Similarly, different jump heights can affect power output (both breaking and propulsion), contact time and leg stiffness (28, 29, 42).

However, our findings suggest that additional verbal instructions, such as “during the landing try to damp the impact at ground contact” (1B and 2B), have the capacity to influence the landing phase (the second peak of the vGRF), independently by the drop height. Our findings clearly demonstrate that trials in which participants were asked to dampen showed a reduced second spike of the vGRF without affecting the performance, in terms of FT or RSI.

In the literature, several studies have focused the attention on kinetic and kinematics behavior of the vGRF-time profile during the braking and propulsion phase (10, 14, 43), providing training information for kinesiologists and practitioners (i.e., how to begin the drop, the feet position, modality of execution, drop height or type of ground contact, etc.) (12, 29, 44). Conversely, few studies have investigated the behavior of the 2nd peak of vGRF during the final landing (45–49). Bates et al. (45, 47) have not found significant differences between the 1st and 2nd peak of vGRF, vice versa in our study a consistent reduction of the second peak force in the vGRF-time profile (about 700 N) if specific verbal instructions are given (1B-2B).

Anyway, anecdotal evidence indicates that there is a lower extremity neuromuscular control deficit during landing (45, 46, 49); in the latter studies (45, 46), the authors highlight that during the 2nd peak of vGRF, the participants show a significant side-to-side asymmetry on kinematics and kinetics parameter (i.e., GRF, joint moment, and knee flexion), indicating that the 2nd ground contact during DVJ, exhibits greater perturbation compared with the 1st. Scarborough et al. (49) report significant differences between the first and second ground contact at the LESS (Landing Error Scoring System) score, which indicates that the 2nd landing was greater than the 1st impact, indicating poorer neuromuscular control. In the same study, differences in LESS scores were also observed between gymnasts and softball players. Even though the LESS score for the second ground contact was significantly higher than that of the first among the two groups, the softball players achieved a superior result in the LESS score. Bates et al. (45, 46) state that in real on-field game situations, basketball players seem to focus their attention more on jumping and how to jump as high as possible to reach a ball or block a pass, and much smaller on the subsequent landing. Considering this, the 2nd peak of vGRF could be a more rigorous parameter and should provide a superior tool to assess the risk factors (45, 46, 48). From this point of view, our results indicate that the use of verbal instructions influences the DVJ's performance appropriately and characterizes the subsequent landing phase dampening the impact with the ground. Etnoyer et al. (4) suggest that the use of verbal instructions in chronic reduce the susceptibility to injury, which allows to structure more safety and controlled patterns during the execution of -landing (the landing is performed without subsequent propulsion phase, in contrast to what happen in the DVJ execution). However, we were unable to find studies evaluating the influence of verbal instructions on the second peak of the vGRF during the DVJ to compare our results.

The use of the DVJ, similar SLST Single-Leg Squat Test (50), as a tool to assess risk behavior for ACL injuries in adolescent female athletes, could have important implications for the future. According to some studies, females exhibit smaller knee flexion angles than males during jump-landing activities (45, 51, 52) and exhibit less knee flexion, coupled with an increase in GRF during landing (2ndGRF) (45, 46). It is argued that during landing, the force exerted on the femorotibial joint immediately following contact results in relative anterior displacement and internal rotation of the tibia, potentially leading to an increase in the injuries to the anterior ligament cruciate (53). According to Shimokochi et al. (54) and Cerulli et al. (55) the magnitude and timing of pressure on the axis of the tibia are correlated and synchronized with the magnitude and timing of GRF. Thus, an increase in vGRF elevates the femorotibial joint pressure, which in turn increases the stress on the ACL. This suggests that vGRF, especially its peak, could be a significant risk factor for the ACL injury. Therefore, the absence of verbal instructions during the execution of DVJs may adversely affect motor control and landing biomechanics related to an increased ACL injury risk (3, 6).

Previous studies (3, 28, 56) have displayed the pattern of muscle activation during the first landing in drop jump revealing that the hamstring muscles were activated before ground contact and reaching their peak of activity (anticipatory activity), while the quadriceps muscle reached its peak of activity after contact. Cowling (3) found that providing a verbal instruction focused on performing the landing (first landing before jumping), the quadriceps muscle activity had a significantly longer duration, than the condition without instruction. This enhancement in agonist/antagonist synergy could be attributed to the fact that verbal instruction enhances the efficacy of inter-muscular recruitment, resulting from an unconscious process (8), possibly triggered by the frontal and parietal cortex (57). From a neuromuscular perspective, the longer muscle activity has been attributed to the need for the quadriceps to control knee flexion during the contact by an eccentric contraction, to prevent the stance limb from “collapsing” under body weight (56). Hence, if the quadriceps muscles exhibit a prolonged period of synchronization with the hamstring, the landing would be more protective for the ACL, as compared to a shorter period (3). Consequently, verbal guidance has the potential to enhance neuromuscular control during the DVJ, thereby potentially reducing the associated ACL injury factors in young girls (3, 4, 58).

The literature indicates that there are differences in kinetic, kinematic, and motor control strategies between individuals of different genders (26, 59, 60). Since women exhibit smaller knee flexion angles at ground contact in comparison to men (61), this may affect the ratio between ground reaction force and center of mass displacement (61). In order to counteract the rate tension development deficit in the hip extensors, women activate the knee extensor earlier than men in a different feedforward control strategy (60). These mechanisms vary depending on the skills and biomechanical constraints related to the type of jump, highlighting different neuromuscular strategies enacted in the two sexes (62), to control the dynamic interactions between the lower limb and the ground. The control strategy provides adequate stiffness differently in the two sexes to safeguard the musculoskeletal system against excessive impact load and to store elastic energy effectively (26).

### 4.1 Limitations

Given that the participants involved were young female adults, our results are applicable to individuals with similar characteristics to those who participated in the present study. In addition, the sample consisted of female athletes from regional levels, therefore the outcomes cannot be generalized to elite athletes, as they may exhibit more structured motor patterns and may be less susceptible to acute verbal instructions. Another limitation concerns the lack of measurements of kinematic parameters such as lower limb joint mechanics and contraction phase timing.

## 5 Conclusion

Our findings suggest that verbal instructions and varying drop heights should be employed when operating the DVJ to enhance performance and mitigate the risk of injury. The parameters of the vGRF-time profile exhibit significant variations during diverse trials in relation to the verbal instructions provided. One noteworthy aspect pertains to the guidance provided for the final landing, which appears to have the potential to enhance safety by reducing the second vGRF peak, while maintaining the required performance during the jump. Attentional focus induced by verbal instruction has a pervasive effect on performance and learning, affecting positively motor control. Before designing a DVJ training program, coaches and/or kinesiologists must first decide what are the goals to provide the athlete with precise and clear verbal instructions for improving specific parameters. In the context of exercise physiology, future research should examine the effect of verbal instruction over time in other populations (i.e., young athletes) to optimize the training process during plyometric training, resulting in specific performance adaptations providing key information on the brain's ability to translate verbal information into coordinated motor actions.

## Data Availability

The raw data supporting the conclusions of this article will be made available by the authors, without undue reservation.
